# 2,3,6-Triphenyl­piperidin-4-one

**DOI:** 10.1107/S1600536809035673

**Published:** 2009-09-12

**Authors:** N. Mahalakshmi Lavanya, R. Anitha, S. Athimoolam, P. Alex Raja, P. L. Nilantha Lakshman

**Affiliations:** a Department of Physics, Kalasalingam University, Krishnan koil 626 190, Tamil Nadu, India; b Department of Organic Chemistry, Madurai Kamaraj University, Madurai 625 021, India; c Department of Food Science and Technology, Faculty of Agriculture, University of Ruhuna, Mapalana, Kamburupitiya 81100, Sri Lanka

## Abstract

In the title mol­ecule, C_23_H_21_NO, the piperidine ring adopts a chair conformation, with the N and carbonyl C atoms as flaps, which deviate on either side of the chair by −0.706 (3) and 0.494 (3) Å, respectively. All three phenyl rings are in equatorial positions on the piperidine ring, making angles with the puckering plane of 73.5 (1), 73.1 (1) and 67.2 (1)°. Though there is no classical hydrogen bonding, the crystal is stabilized by inter­molecular C—H⋯π contacts and π–π stacking inter­actions involving phenyl rings [centroid–centroid distance = 4.424 (2) Å].

## Related literature

For the biological importance of piperidone and its derivatives, see: Robinson (1973 ▶). For similar structures, see: Mobio *et al.* (1989 ▶); Jia *et al.* (1989*a* ▶,*b* ▶); Cheer *et al.* (1984 ▶); Sekar *et al.* (1990 ▶, 1993 ▶); Sukumar *et al.* (1994 ▶); Ompraba *et al.* (2003 ▶). For puckering analysis, see: Cremer & Pople (1975 ▶).
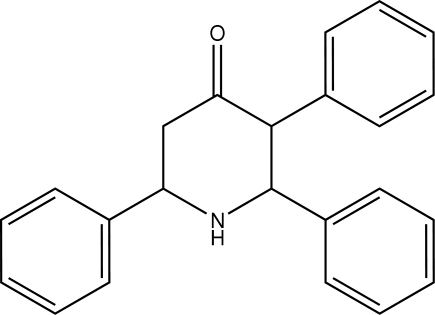


## Experimental

### 

#### Crystal data

C_23_H_21_NO
*M* *_r_* = 327.41
Monoclinic, 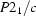

*a* = 12.144 (4) Å
*b* = 5.998 (2) Å
*c* = 25.127 (7) Å
β = 94.55 (2)°
*V* = 1824.3 (9) Å^3^
*Z* = 4
Mo *K*α radiation
μ = 0.07 mm^−1^
*T* = 293 K
0.21 × 0.18 × 0.15 mm

#### Data collection

Nonius MACH3 sealed-tube diffractometer
Absorption correction: ψ scan (North *et al.*, 1968 ▶) *T*_min_ = 0.914, *T*_max_ = 1.000
3552 measured reflections
3193 independent reflections
1906 reflections with *I* > 2σ(*I*)
*R*_int_ = 0.029
3 standard reflections frequency: 60 min intensity decay: <1%

#### Refinement

*R*[*F* ^2^ > 2σ(*F* ^2^)] = 0.038
*wR*(*F* ^2^) = 0.125
*S* = 1.02
3193 reflections
231 parameters
H atoms treated by a mixture of independent and constrained refinement
Δρ_max_ = 0.16 e Å^−3^
Δρ_min_ = −0.16 e Å^−3^

### 

Data collection: *CAD-4 EXPRESS* (Enraf–Nonius, 1994 ▶); cell refinement: *CAD-4 EXPRESS*; data reduction: *XCAD4* (Harms & Wocadlo, 1995 ▶); program(s) used to solve structure: *SHELXTL* (Sheldrick, 2008 ▶); program(s) used to refine structure: *SHELXTL*; molecular graphics: *PLATON* (Spek, 2009 ▶); software used to prepare material for publication: *SHELXTL*.

## Supplementary Material

Crystal structure: contains datablocks global, I. DOI: 10.1107/S1600536809035673/bh2244sup1.cif

Structure factors: contains datablocks I. DOI: 10.1107/S1600536809035673/bh2244Isup2.hkl

Additional supplementary materials:  crystallographic information; 3D view; checkCIF report

## Figures and Tables

**Table 1:** Hydrogen-bond geometry (Å, °)

*D*—H⋯*A*	*D*—H	H⋯*A*	*D*⋯*A*	*D*—H⋯*A*
C14—H14⋯*Cg*2^i^	0.93	3.37	4.111 (4)	139
C35—H35⋯*Cg*3^ii^	0.93	3.37	4.120 (3)	138

Symmetry codes: (i) 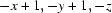; (ii) 
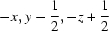
.

**Fig. 1.**
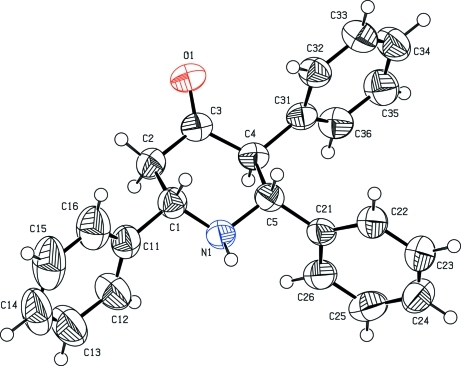
The molecular structure of title compound with atom numbering scheme and 50% probability displacement ellipsoids (Sheldrick, 2008).

**Fig. 2.**
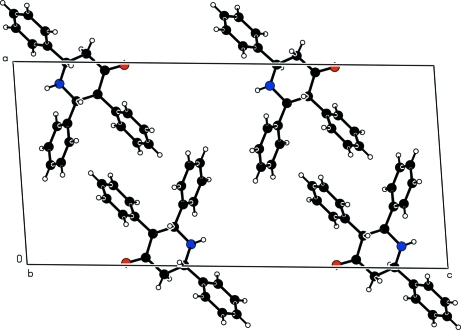
Packing diagram of the molecule viewed down a axis (Sheldrick, 2008).

## supplementary crystallographic information

### Comment

Piperidones possess a variety of biological activities including antihistaminic agents, oral anesthetics, narcotic analgesics, tranquillizers, hypotensive agents, cytotoxic and anti-cancer (Robinson, 1973). Piperidine with 2,6-substitutions have been found to have bactericidal, herbicidal and fungicidal activities (Mobio *et al.*, 1989). The medicinal and fungicidal properties of the piperidones are determined by the nature and position of substituents attached to the ring. Piperidones with different substitutions have been reported earlier (Jia *et al.*, 1989*a*, 1989*b*; Cheer *et al.*, 1984; Sekar *et al.*, 1990, 1993; Sukumar *et al.*, 1994). Crystal and molecular structure of 3-phenylpiperidin-4-one with 2,6-substitution of 4-chlorophenyl was reported, which has similar structural feature of the present compound (Ompraba *et al.*, 2003). In this present investigation, the X-ray crystal and molecular structure of piperidin-4-one with 2,4,6-phenyl substitution is reported.

The configuration and conformation of the title compound, (I), and the atom numbering scheme are shown in the *ORTEP* drawing (Fig. 1). The packing diagram of the title compound is show in Fig. 2. The piperidine ring adopts a chair conformation, with the atoms C1, C2, C4 and C5 in a plane, whereas N1 and C3 deviate by -0.706 (3) and 0.494 (3) Å on either side of this plane. The O1 atom is deviated much from the plane with 1.202 (4) Å. The phenyl rings are planar, with the r.m.s. deviation of 0.0030 Å for ring P1 (C11···C16), 0.0038 Å for ring P2 (C21···C26) and 0.0052 Å for ring P3 (C31···C36). The phenyl rings P1, P2 and P3 make dihedral angles with the piperidine plane, constituted by C1, C2, C4 and C5, of 86.9 (9), 81.9 (9) and 85.5 (8)°, respectively. According to the Cremer & Pople (1975) puckering analysis, the chair conformation of the piperidine ring is confirmed by the amplitude-phase pair of 0.1542 (21) Å and 182.6 (8)° and the single puckering coordinate of -0.5291 (22) Å. The equivalent spherical polar set is 0.5503 (22) Å, 163.9 (2)°, and 182.6 (8)°. The phenyl rings P1, P2 and P3 are in equatorial 2,4,6-positions of the piperidine ring, making an angle with the puckering plane of 73.5 (1), 73.1 (1) and 67.2 (1)°, respectively. The O1 atom is also in equatorial position to the piperdine puckering plane with an angle of 70.5 (1)°. The torsion angles H1—C1—C2—H2A of -173.4 (2)° and H4—C4—C5—H5 of 176.5 (2)° show that the diaxial (*anti*) relationship of the former (6.6°) is deviated much than the later (3.5°) from the ideal value of 180°. The dihedral angles between phenyl rings P1 and P2, P2 and P3 & P3 and P1 are found to be 53.9 (9), 52.1 (8) and 7.6 (2)°, respectively. The C3 atom of the piperdine ring gives short contacts with the O1 atoms of different asymmetric units as C3···O1 (-*x* + 1, + *y* - 1/2, -*z* + 1/2) (3.010 (3) Å) and C3···O1 (-*x* + 1, + *y* + 1/2, -*z* + 1/2) (3.171 (3) Å).

The crystal structure is stabilized through C—H···π and π–π interactions. Two intermolecular C—H···π interactions are observed in the crystal structure with the distances of 4.111 (4) and 4.120 (3) Å to the centroids of the phenyl rings P2 and P3 respectively (Table 1; *Cg*(2) is centroid of phenyl ring C21···C26 and *Cg*(3) is centroid of phenyl ring C31···C36). π–π stacking interactions are observed as intra and intermolecular contacts. As an intramolecular π–π stacking, phenyls rings P2 and P3 are stacked with the centroid to centroid separation of 4.504 (2) Å. Further, the phenyl rings P1 are stacked almost parallel and involved in the π–π interactions around an inversion center (1 - *x*, 1 - *y*, -*z*) with a centroid to centroid separation of 4.424 (2) Å.

### Experimental

Ammonium acetate (0.475 g, 0.0075 mol) was dissolved in ethanol (3 ml) by heating. Benzaldehyde (1.59 g, 0.015 mol) and phenylacetone (1 g, 0.0075 mol) were added to this solution and the mixture heated until the color of the solution changed to yellow. The solution was kept at room temperature for 2-3 days. The solid precipitated was filtered off, washed with ethanol and recrystallized from ethanol and ethyl acetate. The pure compound was obtained in 61% yield.

### Refinement

All C-bonded H atoms were fixed using geometrical constraints and their positions and thermal parameters were refined isotropically riding on the carrier atom. Amine H atom (H1A) was found in a difference map and refined freely. All non-hydrogen atoms are located and refined anisotropically.

### Crystal data

**Table:**

C_23_H_21_NO	*F*(000) = 696
*M**_r_* = 327.41	*D*_x_ = 1.192 Mg m^−^^3^*D*_m_ = 1.173 Mg m^−^^3^*D*_m_ measured by Flotation technique using a liquid mixture of CCl_4_ and xylene
Monoclinic, *P*2_1_/*c*	Mo *K*α radiation, λ = 0.71073 Å
Hall symbol: -P 2ybc	Cell parameters from 25 reflections
*a* = 12.144 (4) Å	θ = 9.7–14.4°
*b* = 5.998 (2) Å	µ = 0.07 mm^−^^1^
*c* = 25.127 (7) Å	*T* = 293 K
β = 94.55 (2)°	Needle, colourless
*V* = 1824.3 (9) Å^3^	0.21 × 0.18 × 0.15 mm
*Z* = 4	

### Data collection

**Table:**

Nonius MACH3 sealed tube diffractometer	1906 reflections with *I* > 2σ(*I*)
Radiation source: fine-focus sealed tube	*R*_int_ = 0.029
graphite	θ_max_ = 25.0°, θ_min_ = 2.3°
ω–2θ scans	*h* = 0→14
Absorption correction: ψ scan (North *et al.*, 1968)	*k* = −1→7
*T*_min_ = 0.914, *T*_max_ = 1.000	*l* = −29→29
3552 measured reflections	3 standard reflections every 60 min
3193 independent reflections	intensity decay: <1%

### Refinement

**Table:**

Refinement on *F*^2^	Secondary atom site location: difference Fourier map
Least-squares matrix: full	Hydrogen site location: inferred from neighbouring sites
*R*[*F*^2^ > 2σ(*F*^2^)] = 0.038	H atoms treated by a mixture of independent and constrained refinement
*wR*(*F*^2^) = 0.125	*w* = 1/[σ^2^(*F*_o_^2^) + (0.0616*P*)^2^ + 0.284*P*] where *P* = (*F*_o_^2^ + 2*F*_c_^2^)/3
*S* = 1.01	(Δ/σ)_max_ < 0.001
3193 reflections	Δρ_max_ = 0.16 e Å^−^^3^
231 parameters	Δρ_min_ = −0.16 e Å^−^^3^
0 restraints	Extinction correction: *SHELXTL* (Bruker, 2000), Fc^*^=kFc[1+0.001xFc^2^λ^3^/sin(2θ)]^-1/4^
Primary atom site location: structure-invariant direct methods	Extinction coefficient: 0.0039 (12)

### Fractional atomic coordinates and isotropic or equivalent isotropic displacement parameters (Å^2^)

**Table:**

	*x*	*y*	*z*	*U*_iso_*/*U*_eq_	
C1	0.99723 (14)	0.4257 (3)	0.12658 (7)	0.0525 (5)	
H1	0.9826	0.5792	0.1374	0.063*	
C2	1.04235 (15)	0.2932 (3)	0.17569 (7)	0.0553 (5)	
H2A	1.0644	0.1463	0.1643	0.066*	
H2B	1.1077	0.3678	0.1917	0.066*	
C3	0.96074 (15)	0.2677 (3)	0.21693 (7)	0.0478 (4)	
C4	0.84239 (14)	0.2127 (3)	0.19584 (6)	0.0463 (4)	
H4	0.8420	0.0553	0.1854	0.056*	
C5	0.80792 (14)	0.3482 (3)	0.14452 (7)	0.0476 (4)	
H5	0.8036	0.5062	0.1541	0.057*	
C11	1.07919 (15)	0.4297 (4)	0.08460 (8)	0.0586 (5)	
C12	1.0872 (2)	0.2564 (5)	0.04918 (9)	0.0847 (8)	
H12	1.0391	0.1361	0.0501	0.102*	
C13	1.1653 (2)	0.2586 (6)	0.01234 (11)	0.1065 (10)	
H13	1.1691	0.1398	−0.0112	0.128*	
C14	1.2362 (2)	0.4296 (8)	0.00998 (12)	0.1083 (12)	
H14	1.2893	0.4287	−0.0147	0.130*	
C15	1.2294 (2)	0.6038 (7)	0.04409 (15)	0.1093 (11)	
H15	1.2775	0.7238	0.0423	0.131*	
C16	1.15128 (19)	0.6046 (5)	0.08169 (11)	0.0870 (8)	
H16	1.1479	0.7245	0.1050	0.104*	
C21	0.69698 (15)	0.2758 (3)	0.11922 (7)	0.0488 (4)	
C22	0.60549 (16)	0.4111 (4)	0.12051 (8)	0.0644 (6)	
H22	0.6126	0.5491	0.1373	0.077*	
C23	0.50388 (18)	0.3465 (5)	0.09750 (9)	0.0797 (7)	
H23	0.4434	0.4406	0.0990	0.096*	
C24	0.49144 (19)	0.1451 (5)	0.07247 (9)	0.0784 (7)	
H24	0.4229	0.1021	0.0567	0.094*	
C25	0.5808 (2)	0.0072 (4)	0.07088 (8)	0.0753 (7)	
H25	0.5728	−0.1305	0.0540	0.090*	
C26	0.68306 (17)	0.0707 (4)	0.09422 (8)	0.0633 (5)	
H26	0.7430	−0.0252	0.0931	0.076*	
C31	0.75856 (15)	0.2369 (3)	0.23659 (6)	0.0476 (4)	
C32	0.74957 (16)	0.4355 (3)	0.26422 (7)	0.0568 (5)	
H32	0.7973	0.5524	0.2582	0.068*	
C33	0.67150 (18)	0.4629 (4)	0.30033 (8)	0.0688 (6)	
H33	0.6674	0.5967	0.3188	0.083*	
C34	0.59921 (19)	0.2924 (4)	0.30919 (9)	0.0733 (6)	
H34	0.5463	0.3105	0.3336	0.088*	
C35	0.60573 (18)	0.0963 (4)	0.28186 (9)	0.0712 (6)	
H35	0.5564	−0.0184	0.2874	0.085*	
C36	0.68516 (16)	0.0677 (3)	0.24617 (8)	0.0597 (5)	
H36	0.6895	−0.0673	0.2283	0.072*	
N1	0.89297 (12)	0.3218 (3)	0.10684 (6)	0.0526 (4)	
O1	0.98843 (11)	0.2814 (2)	0.26421 (5)	0.0572 (4)	
H1A	0.8700 (15)	0.387 (3)	0.0769 (8)	0.060 (6)*	

### Atomic displacement parameters (Å^2^)

**Table:**

	*U*^11^	*U*^22^	*U*^33^	*U*^12^	*U*^13^	*U*^23^
C1	0.0498 (10)	0.0549 (11)	0.0530 (10)	0.0037 (9)	0.0045 (8)	0.0043 (9)
C2	0.0490 (10)	0.0621 (12)	0.0542 (11)	0.0037 (9)	−0.0003 (8)	0.0023 (9)
C3	0.0580 (11)	0.0383 (10)	0.0463 (10)	0.0045 (8)	−0.0016 (8)	0.0025 (8)
C4	0.0552 (10)	0.0401 (9)	0.0434 (9)	0.0022 (8)	0.0021 (8)	−0.0001 (8)
C5	0.0518 (10)	0.0470 (10)	0.0441 (9)	0.0053 (8)	0.0045 (8)	0.0022 (8)
C11	0.0501 (11)	0.0716 (13)	0.0540 (11)	0.0066 (10)	0.0041 (9)	0.0158 (11)
C12	0.0804 (16)	0.104 (2)	0.0731 (15)	0.0042 (14)	0.0276 (13)	−0.0026 (15)
C13	0.0892 (19)	0.154 (3)	0.0812 (18)	0.021 (2)	0.0349 (15)	0.0061 (19)
C14	0.0672 (17)	0.182 (4)	0.0797 (19)	0.038 (2)	0.0269 (14)	0.056 (2)
C15	0.0640 (16)	0.134 (3)	0.133 (3)	−0.0043 (18)	0.0240 (17)	0.058 (2)
C16	0.0665 (14)	0.0945 (19)	0.1012 (19)	−0.0062 (14)	0.0146 (13)	0.0186 (15)
C21	0.0511 (10)	0.0572 (12)	0.0383 (8)	0.0038 (9)	0.0053 (7)	0.0033 (9)
C22	0.0568 (12)	0.0752 (14)	0.0607 (12)	0.0116 (11)	0.0016 (10)	−0.0041 (11)
C23	0.0560 (13)	0.108 (2)	0.0742 (15)	0.0164 (13)	−0.0024 (11)	−0.0066 (15)
C24	0.0578 (13)	0.113 (2)	0.0629 (14)	−0.0084 (14)	−0.0055 (10)	0.0027 (14)
C25	0.0841 (16)	0.0814 (16)	0.0583 (13)	−0.0117 (14)	−0.0066 (11)	−0.0106 (12)
C26	0.0657 (13)	0.0689 (14)	0.0544 (11)	0.0067 (11)	−0.0008 (10)	−0.0082 (11)
C31	0.0548 (10)	0.0473 (11)	0.0402 (9)	0.0003 (9)	−0.0001 (8)	0.0031 (8)
C32	0.0630 (12)	0.0533 (12)	0.0546 (10)	−0.0052 (10)	0.0089 (9)	−0.0037 (9)
C33	0.0781 (14)	0.0658 (14)	0.0642 (13)	0.0040 (12)	0.0161 (11)	−0.0094 (11)
C34	0.0736 (14)	0.0881 (18)	0.0611 (13)	0.0026 (13)	0.0224 (11)	0.0009 (13)
C35	0.0710 (13)	0.0754 (16)	0.0694 (13)	−0.0147 (12)	0.0183 (11)	0.0075 (12)
C36	0.0700 (12)	0.0523 (12)	0.0571 (11)	−0.0078 (10)	0.0061 (10)	0.0001 (10)
N1	0.0505 (9)	0.0654 (11)	0.0419 (8)	0.0059 (8)	0.0034 (7)	0.0056 (8)
O1	0.0705 (8)	0.0531 (8)	0.0463 (7)	−0.0034 (6)	−0.0062 (6)	0.0019 (6)

### Geometric parameters (Å, °)

**Table:**

C1—N1	1.462 (2)	C16—H16	0.9300
C1—C11	1.507 (3)	C21—C22	1.378 (3)
C1—C2	1.532 (3)	C21—C26	1.385 (3)
C1—H1	0.9800	C22—C23	1.376 (3)
C2—C3	1.498 (2)	C22—H22	0.9300
C2—H2A	0.9700	C23—C24	1.365 (4)
C2—H2B	0.9700	C23—H23	0.9300
C3—O1	1.212 (2)	C24—C25	1.367 (3)
C3—C4	1.528 (3)	C24—H24	0.9300
C4—C31	1.507 (2)	C25—C26	1.384 (3)
C4—C5	1.553 (2)	C25—H25	0.9300
C4—H4	0.9800	C26—H26	0.9300
C5—N1	1.464 (2)	C31—C36	1.384 (3)
C5—C21	1.507 (3)	C31—C32	1.387 (3)
C5—H5	0.9800	C32—C33	1.373 (3)
C11—C16	1.372 (3)	C32—H32	0.9300
C11—C12	1.377 (3)	C33—C34	1.377 (3)
C12—C13	1.377 (3)	C33—H33	0.9300
C12—H12	0.9300	C34—C35	1.367 (3)
C13—C14	1.344 (5)	C34—H34	0.9300
C13—H13	0.9300	C35—C36	1.379 (3)
C14—C15	1.358 (5)	C35—H35	0.9300
C14—H14	0.9300	C36—H36	0.9300
C15—C16	1.391 (4)	N1—H1A	0.87 (2)
C15—H15	0.9300		
			
N1—C1—C11	111.81 (15)	C11—C16—C15	120.4 (3)
N1—C1—C2	107.26 (15)	C11—C16—H16	119.8
C11—C1—C2	110.99 (15)	C15—C16—H16	119.8
N1—C1—H1	108.9	C22—C21—C26	117.63 (18)
C11—C1—H1	108.9	C22—C21—C5	121.05 (18)
C2—C1—H1	108.9	C26—C21—C5	121.32 (17)
C3—C2—C1	113.35 (15)	C23—C22—C21	121.5 (2)
C3—C2—H2A	108.9	C23—C22—H22	119.3
C1—C2—H2A	108.9	C21—C22—H22	119.3
C3—C2—H2B	108.9	C24—C23—C22	120.4 (2)
C1—C2—H2B	108.9	C24—C23—H23	119.8
H2A—C2—H2B	107.7	C22—C23—H23	119.8
O1—C3—C2	121.62 (17)	C23—C24—C25	119.3 (2)
O1—C3—C4	122.35 (16)	C23—C24—H24	120.3
C2—C3—C4	115.98 (14)	C25—C24—H24	120.3
C31—C4—C3	114.28 (14)	C24—C25—C26	120.6 (2)
C31—C4—C5	111.20 (14)	C24—C25—H25	119.7
C3—C4—C5	111.06 (14)	C26—C25—H25	119.7
C31—C4—H4	106.6	C25—C26—C21	120.6 (2)
C3—C4—H4	106.6	C25—C26—H26	119.7
C5—C4—H4	106.6	C21—C26—H26	119.7
N1—C5—C21	110.42 (14)	C36—C31—C32	117.72 (17)
N1—C5—C4	108.82 (14)	C36—C31—C4	121.78 (17)
C21—C5—C4	111.87 (15)	C32—C31—C4	120.45 (16)
N1—C5—H5	108.6	C33—C32—C31	121.28 (19)
C21—C5—H5	108.6	C33—C32—H32	119.4
C4—C5—H5	108.6	C31—C32—H32	119.4
C16—C11—C12	117.7 (2)	C32—C33—C34	120.0 (2)
C16—C11—C1	120.6 (2)	C32—C33—H33	120.0
C12—C11—C1	121.7 (2)	C34—C33—H33	120.0
C11—C12—C13	121.0 (3)	C35—C34—C33	119.64 (19)
C11—C12—H12	119.5	C35—C34—H34	120.2
C13—C12—H12	119.5	C33—C34—H34	120.2
C14—C13—C12	121.0 (3)	C34—C35—C36	120.3 (2)
C14—C13—H13	119.5	C34—C35—H35	119.8
C12—C13—H13	119.5	C36—C35—H35	119.8
C13—C14—C15	119.2 (3)	C35—C36—C31	121.0 (2)
C13—C14—H14	120.4	C35—C36—H36	119.5
C15—C14—H14	120.4	C31—C36—H36	119.5
C14—C15—C16	120.7 (3)	C1—N1—C5	111.76 (14)
C14—C15—H15	119.7	C1—N1—H1A	108.1 (13)
C16—C15—H15	119.7	C5—N1—H1A	108.4 (13)
			
N1—C1—C2—C3	−52.9 (2)	N1—C5—C21—C26	51.0 (2)
C11—C1—C2—C3	−175.28 (16)	C4—C5—C21—C26	−70.4 (2)
C1—C2—C3—O1	−140.17 (18)	C26—C21—C22—C23	−0.5 (3)
C1—C2—C3—C4	42.6 (2)	C5—C21—C22—C23	179.96 (18)
O1—C3—C4—C31	15.3 (2)	C21—C22—C23—C24	−0.2 (3)
C2—C3—C4—C31	−167.50 (15)	C22—C23—C24—C25	0.6 (3)
O1—C3—C4—C5	142.07 (17)	C23—C24—C25—C26	−0.2 (3)
C2—C3—C4—C5	−40.7 (2)	C24—C25—C26—C21	−0.5 (3)
C31—C4—C5—N1	179.04 (14)	C22—C21—C26—C25	0.9 (3)
C3—C4—C5—N1	50.59 (19)	C5—C21—C26—C25	−179.60 (18)
C31—C4—C5—C21	−58.7 (2)	C3—C4—C31—C36	−128.49 (18)
C3—C4—C5—C21	172.87 (14)	C5—C4—C31—C36	104.8 (2)
N1—C1—C11—C16	145.40 (19)	C3—C4—C31—C32	54.4 (2)
C2—C1—C11—C16	−94.9 (2)	C5—C4—C31—C32	−72.3 (2)
N1—C1—C11—C12	−36.8 (3)	C36—C31—C32—C33	0.8 (3)
C2—C1—C11—C12	82.9 (2)	C4—C31—C32—C33	178.04 (18)
C16—C11—C12—C13	0.3 (4)	C31—C32—C33—C34	−0.9 (3)
C1—C11—C12—C13	−177.5 (2)	C32—C33—C34—C35	0.0 (3)
C11—C12—C13—C14	0.2 (4)	C33—C34—C35—C36	0.9 (3)
C12—C13—C14—C15	−0.8 (4)	C34—C35—C36—C31	−1.0 (3)
C13—C14—C15—C16	1.0 (4)	C32—C31—C36—C35	0.2 (3)
C12—C11—C16—C15	−0.2 (3)	C4—C31—C36—C35	−177.06 (17)
C1—C11—C16—C15	177.7 (2)	C11—C1—N1—C5	−171.28 (16)
C14—C15—C16—C11	−0.5 (4)	C2—C1—N1—C5	66.8 (2)
N1—C5—C21—C22	−129.47 (18)	C21—C5—N1—C1	170.12 (15)
C4—C5—C21—C22	109.2 (2)	C4—C5—N1—C1	−66.7 (2)

### Hydrogen-bond geometry (Å, °)

**Table:**

*D*—H···*A*	*D*—H	H···*A*	*D*···*A*	*D*—H···*A*
C14—H14···Cg2^i^	0.93	3.37	4.111 (4)	139
C35—H35···Cg3^ii^	0.93	3.37	4.120 (3)	138

Symmetry codes: (i) −*x*+1, −*y*+1, −*z*; (ii) −*x*, *y*−1/2, −*z*+1/2.
